# PCV20 in pediatric pneumococcal prevention: expanded coverage, remaining challenges

**DOI:** 10.3389/fimmu.2025.1707345

**Published:** 2026-01-06

**Authors:** Nicola Principi, Alberto Argentiero, Beatrice Campana, Susanna Esposito

**Affiliations:** 1 Università degli Studi di Milano, Milan, Italy; 2 Pediatric Clinic, University Hospital, Department of Medicine and Surgery, University of Parma, Parma, Italy

**Keywords:** clinical effectiveness, immunogenicity, next-generation vaccines, PCV20, pneumococcal conjugate vaccines, serotype replacement, *Streptococcus pneumoniae*

## Abstract

Pneumococcal conjugate vaccines (PCVs) have substantially reduced the global burden of *Streptococcus pneumoniae* infections in children, yet serotype replacement and variability in immunogenicity continue to challenge long-term effectiveness. The recent introduction of the 20-valent vaccine (PCV20), which adds seven serotypes to those covered by PCV13, represents an important advance as these additional serotypes—such as 8, 10A, 11A, 12F, 15B, 22F, and 33F—are now recognized as significant contributors to invasive and noninvasive pneumococcal disease. To evaluate the potential and limitations of PCV20, we conducted a systematic literature search across PubMed, Scopus, and Web of Science through July 2025, supplemented by manual reference screening, including randomized trials, observational studies, systematic reviews, meta-analyses, and official reports from WHO, CDC, EMA, and FDA. Current evidence indicates that PCV20 elicits broadly noninferior immune responses compared to PCV13, though weaker responses have been observed for specific serotypes, notably 3, 6B, 9V, 19A, and 23F. Mathematical models suggest PCV20 could prevent thousands of additional pneumococcal cases annually compared with PCV13 or PCV15, but other analyzes predict increased breakthrough infections, particularly under reduced-dose regimens. The vaccine’s effectiveness may also be limited by the potential for new serotype replacement, and concerns persist regarding its performance in 2 + 1 or 1 + 1 schedules, with regulatory agencies currently approving only the 3 + 1 regimen. These findings highlight PCV20 as a promising step in pneumococcal prevention but not a definitive solution. Continued surveillance, real-world effectiveness studies, and accelerated development of next-generation higher-valency vaccines will be essential to sustain and expand protection against pneumococcal disease in children.

## 1 Background

*Streptococcus pneumoniae* (Sp), identified more than a century ago, remains a major cause of invasive bacterial diseases (IPD) such as meningitis and septicemia, as well as noninvasive infections (nIPD) including pneumonia and acute otitis media (OMA) (1). The burden of pneumococcal disease disproportionately affects young children, older adults, and individuals with chronic illnesses or immunodeficiencies (2). This epidemiological impact highlighted early on the urgent need for vaccines to mitigate the significant clinical, social, and economic consequences of Sp infections (3).

Initial vaccine development was slow. The first polysaccharide vaccine, containing antigens from 23 serotypes, was introduced in the 1970s but proved ineffective in infants due to the T-cell–independent nature of its immune response (4). A major breakthrough came in the 1990s with conjugate technology, in which capsular polysaccharides were linked to carrier proteins, inducing T-cell–dependent responses effective in young children (5). **Table 1** summarizes the evolution of pneumococcal conjugate vaccine (PCVs) and their serotype coverage. The first conjugate vaccine, PCV7, targeted seven serotypes (4, 6B, 9V, 14, 18C, 19F, 23F) responsible for most IPD cases in the U.S. at the time. Its global introduction led to substantial declines in IPD and nIPD among vaccinated children and also provided indirect protection for older populations through reduced nasopharyngeal carriage and transmission (6).

**Table 1:** Evolution of pneumococcal conjugate vaccines and their serotype coverage.

Vaccine	Year of introduction	Serotypes included	Key features	Limitations
PCV7	2000	4, 6B, 9V, 14, 18C, 19F, 23F	First conjugate vaccine; effective in infants; major reduction in IPD and carriage	Limited serotype coverage; replacement emerged
PCV10	2009	PCV7 + 1, 5, 7F	Broader coverage in children; used in many LMICs	New serotypes emerged (e.g., 19A, 6A)
PCV13	2010	PCV10 + 3, 6A, 19A	Expanded protection against major emerging serotypes; widely adopted globally	Serotype 3 response weak; replacement continued
PCV15	2021	PCV13 + 22F, 33F	Incremental expansion; approved for pediatric and adult use	Limited advantage over PCV13
PCV20	2021–2023	PCV13 + 8, 10A, 11A, 12F, 15B, 22F, 33F	Widest serotype coverage to date; potential to reduce IPD and nIPD	Serotype replacement expected; weaker immunogenicity for some serotypes

IPD, invasive pneumococcal disease; LMICs, low- and middle-income countries; nIPD, noninvasive pneumococcal disease; PCV, pneumococcal conjugate vaccine.

Over time, however, vaccine effectiveness declined, mainly due to the emergence of non-vaccine serotypes—a process known as serotype replacement. Although PCV10 and PCV13 expanded serotype coverage and replaced PCV7, their benefits were eventually offset by the rise of additional pathogenic serotypes. This prompted development of higher-valency formulations, including PCV15 and PCV20. Given its broader serotype spectrum, PCV20 is currently regarded as the most comprehensive option to reduce pneumococcal disease incidence. Several economic and epidemiological analyses predict notable advantages of PCV20 over PCV15, estimating prevention of thousands of additional IPD, pneumonia, and OMA cases, with significant health and financial benefits (7–13).

Nevertheless, emerging evidence suggests that PCV20 may not fully overcome the limitations seen with earlier vaccines. Concerns remain regarding its serotype coverage, immunogenicity, and potential for new serotype replacement, which could limit long-term effectiveness. The objective of this narrative review is to critically examine the limitations of PCV20 and identify challenges that must be addressed to optimize pneumococcal disease prevention in children.

## 2 Methods

The literature review was conducted to identify studies addressing the prevention of Sp infections through polysaccharide conjugate vaccines. Searches were performed in PubMed, Scopus, and Web of Science, covering all records from database inception to July 2025. The strategy combined Medical Subject Headings (MeSH) and free-text terms, linked with Boolean operators (AND, OR), to maximize sensitivity and specificity. Search terms included: “*Streptococcus pneumoniae*,” “pneumococcus,” “pneumococcal conjugate vaccines,” “PCV7,” “PCV13,” “PCV20,” “invasive infections,” “acute otitis media,” “pneumonia,” “epidemiology,” “infants,” and “children.” Original articles and abstracts including randomized controlled trials, and critical reviews including systematic review and meta-analysis were selected and examined. Trials outside the scope of this review were not considered. References of the retrieved articles were also screened to search for potentially relevant papers.

Study selection was independently conducted by two reviewers (AA and BRC), who screened titles and abstracts for relevance. Disagreements were resolved by consensus or, when necessary, adjudication by a third reviewer (SE). For included studies, data extraction covered publication details (authors, year, country), study design, population characteristics, and outcomes related to pneumococcal conjugate vaccine use in infants and children.

## 3 Results

### 3.1 Serotype coverage

PCV20 builds upon existing serotypes in PCV13 (1, 3, 4, 5, 6A, 6B, 7F, 9V, 14, 18C, 19A, 19F, 23F) and includes seven additional serotypes (8, 10A, 11A, 12F, 15B, 22F, 33F). These additional serotypes were selected to address the growing burden of IPD, nIPD, and antibiotic-resistant infections observed after widespread PCV13 use. Current evidence suggests that PCV20 largely meets emerging epidemiological needs and could substantially enhance pneumococcal disease control (14, 15).

A systematic review of 127 studies conducted between 2010 (the year of PCV13 introduction) and August 2020 across the Americas, Europe, and Western Pacific regions found that non-PCV13 serotypes included in PCV20 accounted for 27.8% of overall IPD cases and 28.6% of acute otitis media (AOM) cases in children (16). Within this group, serotypes 10A and 15B/C—unique to PCV20—were most frequently associated with both invasive and noninvasive disease, often linked to severe clinical presentations. Serotype 15B/C also showed frequent resistance to penicillin and macrolides.

A second systematic review of 58 pediatric studies conducted between 2010 and 2021 across WHO regions (AMRO, AFRO, EMRO, SEARO, WPRO) confirmed similar findings (17). The five most prevalent serotypes during the PCV13 period were 5, 12F, 15B/C, 19A, and 33F, underscoring that, in several countries with high circulation of serotypes exclusively included in PCV20, only this vaccine could offer sufficient protection.

Evidence from carriage studies further highlights the importance of PCV20. In Portugal, Candeias et al. (18) reported that by 2018–2020, non-PCV13 serotypes represented 89.3% of nasopharyngeal isolates in children, with PCV20-exclusive serotypes (15B/C, 10A, 11A) comprising nearly one-third. Similarly, Tiley et al. (19) showed in England that three of the five most frequently isolated serotypes post-PCV13 introduction (15B/C, 10A, 11A) were covered only by PCV20. These findings suggest diminishing herd immunity under PCV13, but potential restoration with PCV20.

Despite these advantages, past experience with PCV7 and PCV13 suggests that widespread use of PCV20 may eventually trigger new serotype replacement, with global vaccine effectiveness declining after 3–4 years. Once diseases caused by non-vaccine serotypes exceed those caused by vaccine serotypes, overall protection conferred by conjugate vaccines wanes. This trend also diminishes herd immunity in older populations. Indeed, such dynamics have already been documented. In Germany, the maximum impact of PCV13 on overall IPD incidence (–48%) occurred in 2012/13, followed by a rebound to –26% in 2015/16, with non-PCV13 serotypes accounting for more than 80% of cases (20). In England and Wales, complete nasopharyngeal replacement was evident within two years of PCV13 introduction, with a subsequent rise in non-PCV13 IPD cases among children under five years (21).

If similar replacement occurs under PCV20, its maximum benefits may be short-lived, necessitating development of broader-valency formulations. Next-generation PCVs are already under development. PCV24, currently in phase 2 trials, extends PCV20 coverage with four additional serotypes (2, 9N, 17F, 20), while PCV31 adds further coverage (7C, 15A, 16F, 23A, 23B, 31, 35B), potentially protecting against ~94% of IPD in U.S. children under five (22–24). Health-economic analyses estimate an annual burden of disease covered by PCV24 serotypes of $1.3 billion and $7.5 billion for those addressed by PCV31, reinforcing the importance of serotypes not targeted by PCV20 (25).

Taken together, these findings demonstrate that while PCV20 significantly expands serotype coverage and may reduce disease burden in the short to medium term, its long-term effectiveness will depend on continuous epidemiological surveillance and timely development of higher-valency PCVs.

### 3.2 Immunogenicity

Evaluation of immunogenicity is essential for newly developed PCVs, since their potential effectiveness is primarily inferred from the immune responses they generate. The first conjugate vaccine, PCV7, was licensed after extensive randomized controlled trials demonstrating safety and efficacy (26). However, subsequent higher-valency PCVs could not be assessed with similar trial designs because of practical and ethical constraints. Placebo-controlled studies were no longer acceptable, as withholding an effective vaccine would expose participants to preventable disease. As a result, vaccine performance has increasingly been inferred through correlates of protection, immunological markers that approximate clinical effectiveness.

Based on analyses of PCV7, an IgG anticapsular antibody concentration ≥0.35 µg/mL one month after primary immunization was proposed as a correlate of protection against IPD (27). Yet, this threshold is an imperfect surrogate. Results vary depending on laboratory methodology, population characteristics, and serotype-specific immune responses. Importantly, it does not account for herd immunity effects, which are central to the overall impact of PCVs. Evidence suggests that higher antibody levels are required for effective protection against certain serotypes (e.g., 2.83 µg/mL for serotype 3; 1.17 µg/mL for serotype 19F) (28) and for preventing nonbacteremic pneumonia (29), otitis media (30), or nasopharyngeal carriage (31). Consequently, broader immunological assessments are now recommended, including IgG response rates, geometric mean concentrations (GMCs), and opsonophagocytic activity (OPA), evaluated both after primary series and booster doses (32).

WHO guidelines stipulate that noninferiority (NI) of immune responses for new PCVs can be demonstrated by either IgG response rates or GMCs. Meeting NI for every serotype is not mandatory, as some trials instead require that a predefined number of serotypes satisfy NI criteria (33).

Concerns regarding immunogenicity have been raised with increasing vaccine valency. Comparative studies suggest that broader serotype coverage can compromise responses to individual serotypes, possibly reducing overall effectiveness, particularly against nIPD and colonization (34). Contributing mechanisms include immune interference, competition among B cell populations, and carrier-induced epitope suppression (35–38). For instance, PCV13 showed slightly reduced responses to certain serotypes compared to PCV7 under the 3 + 1 schedule, though OPA titers remained robust and licensure was granted (39–41).

PCV20 has been evaluated under both 3 + 1 and 2 + 1 schedules (**Table 2**). In the 3 + 1 regimen, after the third dose, IgG GMCs were generally noninferior to PCV13 for shared serotypes, though lower responses were observed for serotypes 1, 3, 4, 9V, and 23F. OPA titers were slightly reduced but improved following the booster, resulting in overall comparable immune responses to PCV13. These findings supported approval of PCV20 for use in the 3 + 1 schedule in the U.S (42, 45). and later in the EU (46).

**Table 2:** Summary of PCV20 immunogenicity characteristics.

Schedule	Primary outcome	Serotypes meeting NI criteria	Serotypes with reduced response	Booster effect	Regulatory status
3 + 1 (42)	IgG GMCs and OPA	Most common serotypes; strong overall NI	1, 3, 4, 9V, 23F (lower responses)	Differences resolved post-booster	Approved (USA, EU)
2 + 1 (43)	IgG GMCs and OPA	NI met for 16 of 20 serotypes	1, 3, 4, 5, 6A, 6B, 9V, 18C, 23F	Most differences resolved after booster, except 6B and 3	Not approved (FDA, EMA)
1 + 1 (44)	Limited data	—	Concerns raised from PCV13 experience	—	Not recommended

GMC, geometric mean concentrations; NI, noninferiority; OPA, opsonophagocytic activity; PCV, pneumococcal conjugate vaccine.

By contrast, studies of the 2 + 1 schedule raised greater concerns. One month after dose 2, 16/20 vaccine serotypes met NI criteria for PCV20 to PCV3 for ≥1 of 2 primary immunogenicity objectives (IgG geometric mean concentrations and percentages of participants with predefined serotype-specific IgG concentrations). Of the matched serotypes, 6A, 6B, 9V and 23F missed NI criteria for both objectives. Two of the 7 additional serotypes (10A, 12F) missed the statistical NI criterion only for the percentage of participants with predefined IgG concentrations (43). After dose 3, residual gaps remained for serotypes 6B and 3, although OPA titers were significantly increase compared to those observed after those 2. This was particularly notable for the serotypes missing statistical NI criteria 1 month after the primary vaccine series. Given that IPD is most severe in the first six months of life, this weaker early protection prompted the FDA (47) and EMA (48) to restrict PCV20 authorization to the 3 + 1 schedule. Adoption of this regimen, however, introduces added costs and logistical challenges for pediatric immunization programs. Some experts have nonetheless supported the use of PCV20 in a 2 + 1 schedule, as seen in Canada (44), noting that this approach provides notable public health and logistical benefits. These include reduced program expenses and the possibility of increased vaccine uptake, ultimately resulting in better protection for the pediatric population. On the other hand, for economic considerations and to decrease the total number of vaccines administered to children, a 2 + 1 PCV schedule is now accepted as a standard of care for healthy children by the WHO (49).

Further concerns relate to the 1 + 1 schedule currently used for PCV13 in the UK. Recent evidence suggests higher IPD rates under 1 + 1 compared with the former 2 + 1 regimen (50). Dynamic modeling has predicted that a shift from 2 + 1 to 1 + 1 with PCV20 would similarly increase IPD burden (51).

Overall, PCV20 demonstrates strong immunogenicity under the 3 + 1 schedule but more limited responses in reduced-dose regimens. These findings highlight the delicate balance between extending serotype coverage and maintaining adequate immunological protection, particularly in early infancy.

### 3.3 Clinical effectiveness

Although PCV20 demonstrates immunogenicity comparable to PCV13, this does not necessarily ensure equivalent clinical efficacy. The use of NI margins permits small differences in antibody responses, which may translate into reduced protection for serotypes that are inherently less immunogenic, such as 3, 7F, 19A, and 19F. Similar concerns were raised previously when PCV13 showed weaker responses for certain serotypes compared with PCV7. Consequently, relying on PCV7 and PCV13 as reference comparators for immunobridging studies of PCV20 could potentially lower overall protection levels and shorten duration of immunity.

The definitive assessment of PCV20 effectiveness will only be possible through post-licensure data from large-scale implementation. In the meantime, mathematical modeling provides important insights, though results are not entirely consistent. Most models predict substantial advantages of PCV20 over earlier vaccines. However, some raise concerns. For example, Bakker et al. (52) modeled the impact of routine PCV20 vaccination in France and found that, for infants under 12 months, breakthrough IPD cases caused by PCV13 serotypes increased by 350% under a 2 + 1 schedule and by 65% under a 3 + 1 schedule.

Similarly, Ryman et al. (53) modeled predicted vaccine effectiveness by applying serotype-specific protective antibody thresholds derived from PCV7 and PCV13 studies to PCV20 serotypes. Their results suggested PCV20 effectiveness ranged from 74–99% for PCV7 serotypes and 64–92% for PCV13-nonPCV7 serotypes. PCV20’s predicted performance matched PCV7 for four shared serotypes (9V, 18C, 19F, 23F) and PCV13 for three shared serotypes (6A, 7F, 35). However, lower effectiveness was predicted for serotypes 4, 6A, and 14 compared with PCV7, and for serotypes 1, 3, and 19A compared with PCV13.

These findings must be interpreted cautiously. Modeling studies have inherent limitations (54–56). Results are often not generalizable beyond trial populations because immune responses vary by age, genetics, environment, and sampling timing. Many models rely solely on antibody concentrations, without incorporating opsonophagocytic activity (OPA), which plays a critical role in protection. In addition, vaccine effectiveness in real-world settings is influenced by programmatic factors such as vaccine uptake, coverage, herd immunity effects, and evolving serotype epidemiology (57).

Taken together, while negative predictions highlight potential vulnerabilities, they do not definitively undermine PCV20’s utility. Rather, they underscore the need for continuous post-implementation evaluation of serotype circulation and the incidence and severity of IPD and nIPD. Only through rigorous surveillance can the true effectiveness of PCV20 be determined and strategies adapted to sustain long-term protection.

## 4 Discussion

The introduction of PCV20 marks a pivotal development in pneumococcal prevention, particularly for pediatric populations (**Table 3**). By expanding serotype coverage to include 20 strains—adding serotypes 8, 10A, 11A, 12F, 15B, 22F, and 33F—PCV20 directly addresses pathogens that have emerged as dominant causes of invasive pneumococcal disease (IPD) and noninvasive infections in the post-PCV13 era. This broad coverage has significant implications not only for reducing disease incidence but also for mitigating antimicrobial resistance, as several of the added serotypes are frequently resistant to first-line antibiotics.

**Table 3:** Key opportunities and limitations of PCV20 in childhood pneumococcal prevention.

Domain	Opportunities	Limitations
Serotype coverage	Broader than PCV13; includes emerging serotypes (8, 10A, 11A, 12F, 15B, 22F, 33F)	Still excludes relevant serotypes (e.g., 24F, 23B, 35B); risk of future replacement
Immunogenicity	Generally noninferior to PCV13; strong OPA response after booster	Lower responses to serotypes 3, 6B, 9V, 19A, 23F; potential reduced protection with 2 + 1 schedule
Clinical effectiveness	Models predict significant reductions in IPD, pneumonia, and AOM	Some models suggest increased breakthrough infections under certain schedules
Programmatic use	Can further reduce childhood disease burden; indirect protection expected	Approved only for 3 + 1 schedule in US/EU; added cost/logistics compared to 2 + 1
Future directions	Basis for next-generation vaccines (PCV24, PCV31); potential near-complete IPD coverage	Continuous surveillance required; serotype replacement inevitable

IPD, invasive pneumococcal disease; PCV, pneumococcal conjugate vaccine.

A central strength of PCV20 lies in its alignment with contemporary epidemiology. Surveillance data demonstrate that serotypes now incorporated into PCV20 account for a substantial proportion of pediatric IPD and acute otitis media cases. (58) By targeting these serotypes, PCV20 is positioned to restore vaccine effectiveness that had declined due to serotype replacement under PCV13. Another strength is its strong immunogenicity profile under the 3 + 1 regimen, where most shared serotypes achieve noninferior responses compared to PCV13, supported by robust opsonophagocytic activity. This provides reassurance of protective efficacy, particularly after the booster dose, when responses become comparable to or exceed those of earlier vaccines. In addition, PCV20 carries clear health-economic advantages. Modeling studies consistently predict that transitioning from PCV13 to PCV20 could prevent thousands of cases of IPD, pneumonia, and otitis media annually, yielding substantial cost savings through reduced hospitalizations, antibiotic use, and indirect effects such as decreased parental work absence. Lastly, PCV20 serves as a stepping stone to next-generation PCVs. Candidates such as PCV24 and PCV31 aim to close remaining coverage gaps by including additional serotypes, moving closer to near-complete protection. Thus, PCV20 represents both an immediate public health benefit and a foundation for future innovation.

Despite its promise, PCV20 faces several important limitations. While generally noninferior to PCV13, PCV20 produces weaker immune responses against several clinically significant serotypes, including 3, 6B, 9V, 19A, and 23F. These gaps are concerning given the high burden of disease caused by these serotypes, which have historically been difficult to control. Reduced responses may compromise protection during early infancy, when children are most vulnerable. Regulatory authorities currently endorse PCV20 only in the 3 + 1 regimen, reflecting concerns about insufficient immunogenicity under reduced-dose schedules. This poses programmatic and economic challenges, especially in low- and middle-income countries, where streamlined schedules are crucial for feasibility. The lack of flexibility may hinder widespread adoption, delaying potential public health benefits. Experience with earlier PCV shows that the selective pressure exerted by PCV20 is likely to promote expansion of non-vaccine serotypes. Emerging serotypes not included in PCV20 are already contributing significantly to IPD in some regions. Without ongoing surveillance and rapid adaptation of vaccine formulations, these dynamics threaten to erode long-term effectiveness and diminish herd protection. Moreover, to date, most assessments of PCV20 effectiveness derive from immunogenicity bridging studies and predictive modeling. While valuable, these methods cannot fully account for real-world complexities such as population heterogeneity, co-administered vaccines, variable uptake, or region-specific serotype prevalence. Only large-scale post-licensure data will clarify its true protective impact, duration of immunity, and capacity to generate herd effects. Furthermore, the higher cost and dosing requirements of PCV20 raise concerns about global health equity. Low- and middle-income countries, which bear a disproportionate burden of pneumococcal disease, may face delayed access due to financial and logistical barriers. If implementation lags behind high-income countries, global disparities in disease burden may widen. PCV20 should therefore be viewed as a transitional solution rather than a definitive endpoint. Continued epidemiological monitoring is essential to identify shifts in serotype distribution, breakthrough infections, and potential vaccine failures. Integration of PCV20 into immunization programs will also require adaptive strategies that balance broad coverage with cost-effectiveness, particularly in resource-limited settings.

Looking ahead, next-generation vaccines such as PCV24 and PCV31 offer the prospect of near-complete serotype coverage and may help preempt replacement dynamics. Beyond polysaccharide conjugates, protein-based pneumococcal vaccines are under investigation, aiming to induce serotype-independent protection by targeting conserved pneumococcal antigens. Such innovations could ultimately complement or supersede conjugate vaccines, offering more sustainable control of pneumococcal disease.

## 5 Conclusion

PCV20 represents an important milestone in the fight against pneumococcal disease, offering broader protection than previous conjugate vaccines and promising to reduce the burden of invasive and noninvasive infections in children worldwide. However, its ultimate success will depend on overcoming several challenges, including suboptimal responses to certain serotypes, limitations under reduced-dose schedules, and the inevitability of serotype replacement. To maximize its benefits, careful integration into national immunization programs, coupled with sustained epidemiological surveillance and real-world effectiveness studies, is essential. At the same time, investment in next-generation higher-valency vaccines, such as PCV24 and PCV31, remains critical to address remaining gaps and ensure long-term protection. In this evolving landscape, PCV20 should be viewed as a meaningful step forward rather than a definitive solution, marking progress in a broader continuum of pneumococcal vaccine innovation.
